# Supplementary figures and images for: Protein Connectivity in Chemotaxis Receptor Complexes
Source: PLoS Comput Biol. 2015 Dec 8;11(12):e1004650. doi: 10.1371/journal.pcbi.1004650 (PMC4672929; doi:10.1371/journal.pcbi.1004650)

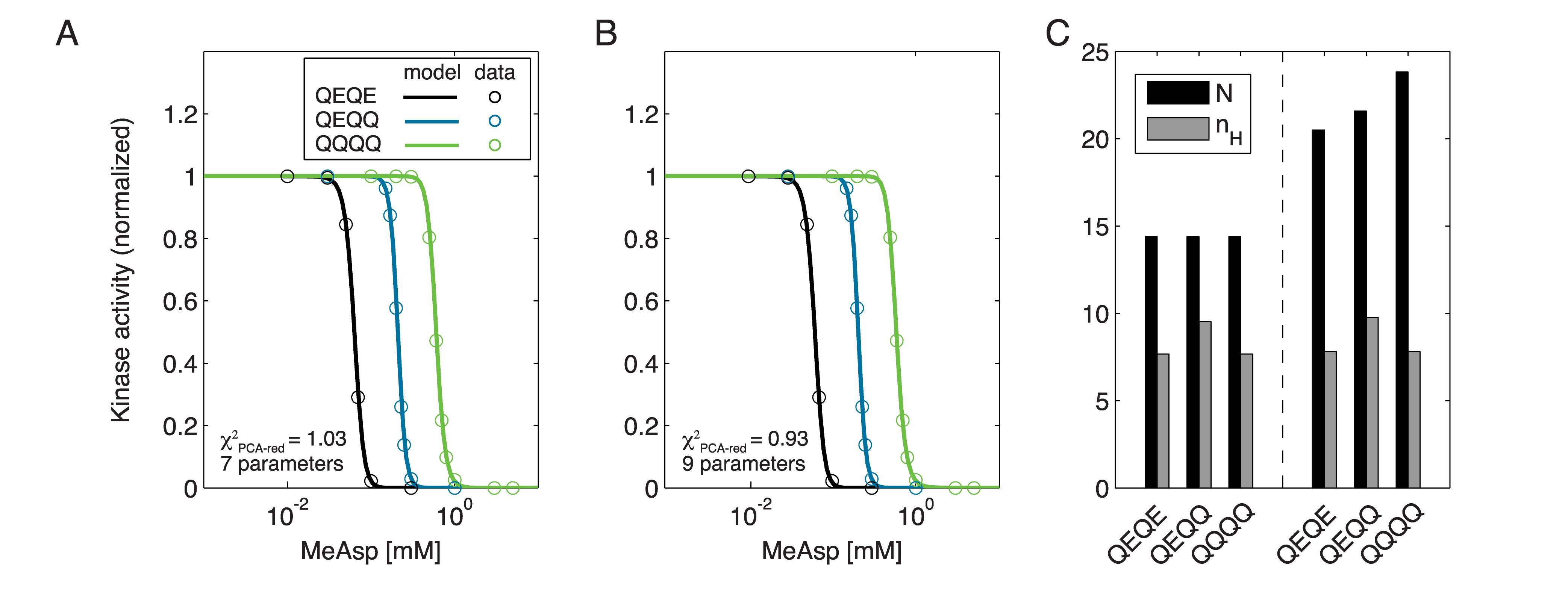

Supplement: S1 Fig — (A,B) Kinase activity for Tar receptors in QEQE (black), QEQQ (blue) and QQQQ (green) modification states fitted in the classical MWC model with (A) constant and (B) variable receptor-complex size N. The fitting based on Principal Component Analysis (PCA) follows Ref. [23]. Relating the resulting χ 2 values to the degrees of freedom, here calculated as the number of included PCA components minus the number of model parameters, results in similar goodness-of-fit values χPCA-red2 with subscript ‘red’ describing the reduced χ 2. However, it should be noted that the actual χPCA-red2 here is rather a supportive argument to the apparent similarity of both fits, as the number of degrees of freedom is not well defined for nonlinear models [57]. (C) Comparison of receptor-complex size N and Hill coefficient n H for fits with constant N (left, panel A) and variable N(m) (right, panel B). (TIF) [file pcbi.1004650.s001.tif]

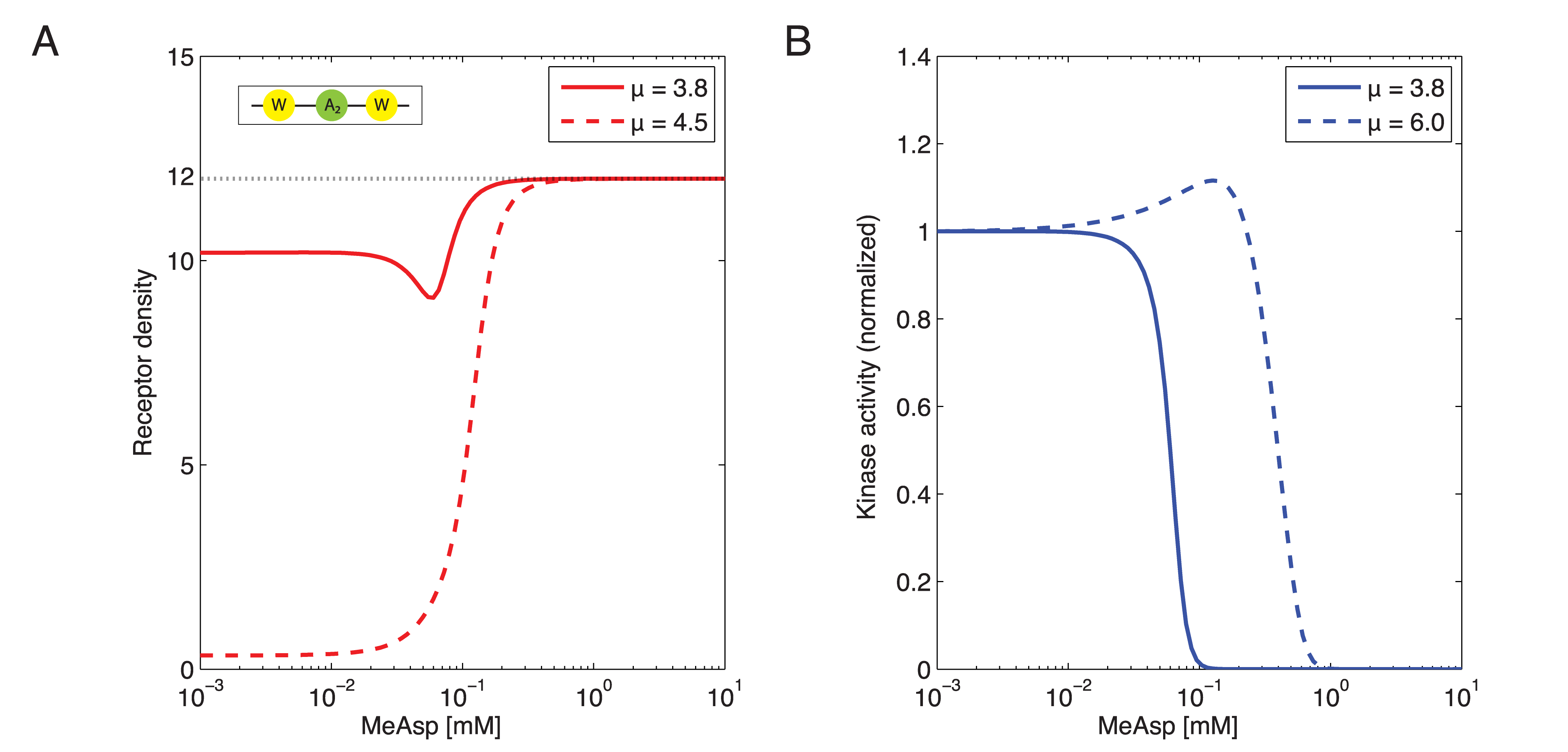

Supplement: S2 Fig — (A) Receptor density as a function of ligand concentration for μ = 3.8 (solid) and μ = 4.5 (dashed). For a constant chemical potential μ, the values of the single dimer energies f on and f off Eq (1) decrease with increasing ligand concentration c. The decrease in the resulting complex energies F on and F off Eq (4) is stronger for larger complexes. Hence, larger complexes are favored with increasing ligand concentration Eq (7), resulting in an increased receptor density Eq (11). The interim decrease in ρ for μ = 3.8 is the result of an ensemble effect. While the probabilities of all complex sizes increase with c, the increase for larger complexes starts at higher c values. Starting off at a smaller receptor density, this effect is not visible for μ = 4.5. Finally both densities asymptotically approach the maximal value of 12. (B) Normalized kinase activity as a function of ligand concentration for μ = 3.8 (solid) and μ = 6.0 (dashed). In the case of μ = 3.8, the increase in receptor density is not apparent in the dose-response curve as the receptors ‘turn off’ before the density increase comes into effect. For μ = 6.0, however, the increase in receptor density yields a ‘bump’ in the dose-response curve. All plots were generated using the same parameters for QEQE as in Fig 2. (TIF) [file pcbi.1004650.s002.tif]

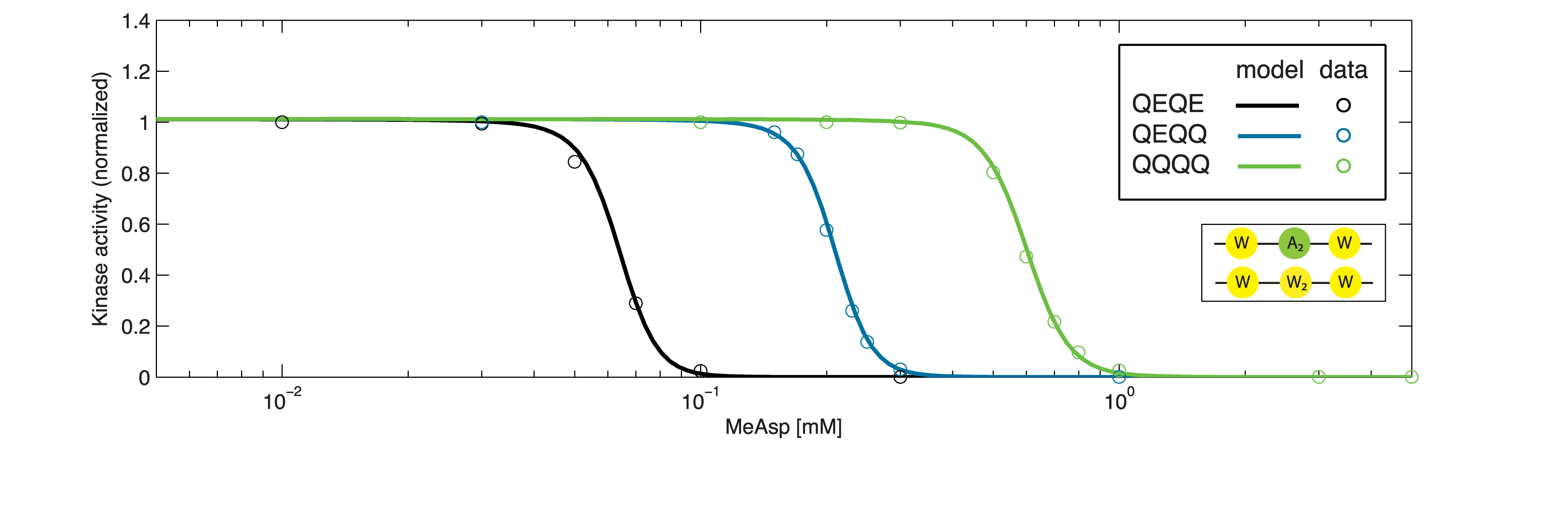

Supplement: S3 Fig — Plot following Fig 2A showing kinase activity for Tar receptors in QEQE (black), QEQQ (blue) and QQQQ (green) modification states. Here the model includes both linkers (–CheW–CheW2–CheW– and –CheW–CheA2–CheW–). For simplicity parameters are the same as in Fig 2 with the additional value for μ W2 in agreement with the value used in Fig 8. (TIF) [file pcbi.1004650.s003.tif]

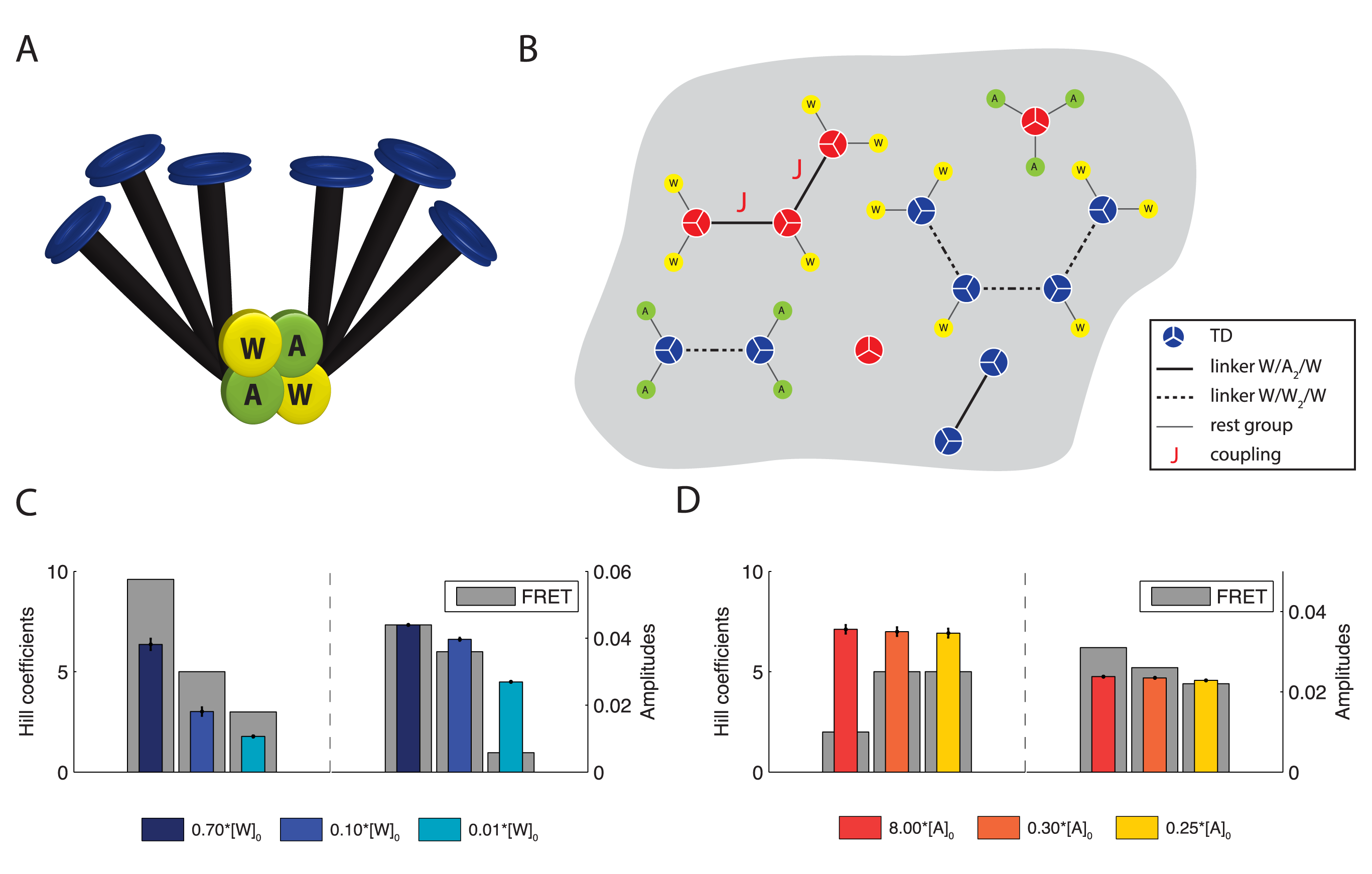

Supplement: S4 Fig — (A) Schematics of an alternative linker =CheW/CheA2/CheW= with both CheA and CheW contacting the trimers directly. (B) Exemplary ensemble of complexes in a membrane. The two linkers are represented by solid (=CheW/CheA2/CheW=) and dashed (=CheW/CheW2/CheW=) lines. Active and inactive TDs are shown in red and blue, respectively. Each linker between active TDs contributes a coupling energy J. As monomeric CheA binds directly to trimers, all linker molecule energies are indicated for monomers, hence the linker energy contributions in Eq (4) become (x − 1)(2μ W + 2μ A) (standard linker) and (x − 1)(2μ W + 2μ W) (CheW-only linker). (C,D) In analogy to Fig 8, we fitted the alternative model to the experimental data for varied expression levels of CheW and CheA using a global optimization routine (see Materials and Methods). While the alternative model is qualitatively able to describe the effect of changing CheW levels correctly (C), it falls short of reproducing the cooperativity decrease for increasing CheA levels (D) with nearly identical curves as best fitting result. Model parameters: Δϵ(QEQE) = −0.23, KD,Tsron=17.78, KD,Tsroff=0.02, ρ W = 2.57, ρ A = 3.96, μW0=-1.86, μA0=-3.82 and J = −3.99. (TIF) [file pcbi.1004650.s004.tif]
